# Accelerated Carbonation of Vibro-Compacted Porous Concrete for Eco-Friendly Precast Elements

**DOI:** 10.3390/ma16082995

**Published:** 2023-04-10

**Authors:** Antonio Manuel Merino-Lechuga, Ágata González-Caro, Enrique Fernández-Ledesma, José Ramón Jiménez, José María Fernández-Rodríguez, David Suescum-Morales

**Affiliations:** 1 Área de Ingeniería de la Construcción, E.P.S de Belmez, Universidad de Córdoba, 14240 Córdoba, Spain; ammlechuga@uco.es (A.M.M.-L.); efledesma@uco.es (E.F.-L.); p02sumod@uco.es (D.S.-M.); 2 Área de Química Inorgánica, E.P.S de Belmez, Universidad de Córdoba, 14240 Córdoba, Spain; q32gocaa@uco.es

**Keywords:** construction and demolition waste, porous concrete, CO_2_ uptake, accelerated carbonation

## Abstract

This research studied the effect of accelerated carbonation in the physical, mechanical and chemical properties of a non-structural vibro-compacted porous concrete made with natural aggregates and two types of recycled aggregates from construction and demolition waste (CDW). Natural aggregates were replaced by recycled aggregates using a volumetric substitution method and the CO_2_ capture capacity was also calculated. Two hardening environments were used: a carbonation chamber with 5% CO_2_ and a normal climatic chamber with atmospheric CO_2_ concentration. The effect of curing times of 1, 3, 7, 14 and 28 days on concrete properties was also analysed. The accelerated carbonation increased the dry bulk density, decreased the accessible porosity water, improved the compressive strength and decreased the setting time to reach a higher mechanical strength. The maximum CO_2_ capture ratio was achieved with the use of recycled concrete aggregate (52.52 kg/t). Accelerate carbonation conditions led to an increase in carbon capture of 525% compared to curing under atmospheric conditions. Accelerated carbonation of cement-based products containing recycled aggregates from construction and demolition waste is a promising technology for CO_2_ capture and utilisation and a way to mitigate the effects of climate change, as well as promote the new circular economy paradigm.

## 1. Introduction

Since the mid-19th century, the rise of the economy and industry has grown exponentially. This has led to an increase in pollution and greenhouse gas (GHG) emissions, and soon the scarcity of raw materials and natural resources will be more evident [1]. For this reason, one of the main global challenges today is the preservation of the planet, which includes sustainable use of natural resources and raw materials, the reuse of waste for new production processes (circular economy) and the mitigation of climate change. The construction sector has contributed to these problems, being one of the industries with the greatest impact on the environment due to the massive consumption of non-renewable natural resources such as aggregates and the emission of CO_2_ into the environment derived from both the production of Portland cement and transportation of materials. During the construction, use, maintenance and subsequent demolition of building and infrastructures, large amounts of construction and demolition waste (CDW) are produced [2,3,4,5,6,7] that must be properly managed.

Carbon dioxide (CO_2_) is the main greenhouse gas and is one of the main causes of global warming [8,9,10]. Up to 55% of greenhouse gas (GHG) emissions correspond to CO_2_. In the construction sector, the manufacture of building materials generates approximately 73% of carbon, of which the manufacture of Portland cement accounts for 41%, hence the scientific and social alarm around the carbon footprint of building materials and their influence on climate change [10]. In concrete production, it is estimated that 0.2 to 0.4 tonnes of CO_2_ equivalent are produced per cubic metre of concrete [11,12]. Globally, the total cement production was 4.6 billion tonnes in 2015, increasing at a rate of 2.5% per year [13]. Concrete is the most widely used building material in the world, estimated to be used five times more by weight than steel, and in some countries this ratio can even be as high as 10 to 1 [14], due to the fact that in both civil and building structures the concrete sector is much larger than that of steel and even in non-structural precast elements the use of steel is not necessary. As a positive aspect, concrete and cement-based materials have the capacity to fix atmospheric CO_2_ irreversibly through the process of cement carbonation [15].

Concrete carbonation is a natural process affected by natural exposure conditions. The progress of carbonation depends mainly on the permeability of concrete, the relative humidity (RH) content and the concentration of CO_2_ available in the environment. When concrete is exposed to the CO_2_ environment, the gas penetrates into it, mainly through pores and microcracks. Once there, the carbon dioxide reacts with the primary hydration products (e.g., calcium hydroxide Ca(OH)_2_ or CSH), resulting in calcium carbonate CaCO_3_ and silica gel, as shown in Equations (1) and (2) [13,16,17,18,19,20,21]. Atmospheric CO_2_ can also react with calcium aluminate hydrate to form CaCO_3_, according to Equation (3) [13,17,18,19,20,21]. In addition, non-hydrated cement clinkers of cementitious materials, such as tricalcium silicate (C_3_S), also called alite, or dicalcium silicate (C_2_S), also called belite, can further react with CO_2_, as shown in Equations (4) and (5), respectively [13,21,22,23].

(1) $${Ca\left(OH\right)}_{2}+{CO}_{2}\to {CaCO}_{3}+H_{2}O$$

(2) $$C-S-H+{CO}_{2}\to {CaCO}_{3}+{SiO}_{2}\cdot \mu H_{2}O$$

(3) $$4Cao\cdot {Al}_{2}O_{3}\cdot 13H_{2}O+4{CO}_{2}\to 4{CaCO}_{3}+2Al{\left(OH\right)}_{3}+10H_{2}O$$

(4) $$C_{3}S+(3-x){CO}_{2}+yH_{2}O\to C_{x}SH_{y}+(3-x){CaCO}_{3}$$

(5) $$C_{2}S+(2-x){CO}_{2}+yH_{2}O\to C_{x}SH_{y}+(2-x){CaCO}_{3}$$

The carbonation phenomenon is based on atmospheric CO_2_ concentration, so in order to achieve accelerated carbonation, special curing conditions with a high concentration of CO_2_ must be used [13,16,24]. Other factors that affect the carbonation process are atmospheric pressure, temperature, humidity and the chemical composition of the cement [20]. As the amount of clinker increases, the amount of CO_2_ absorbed increases, but the greater the amount of cement per cubic metre of concrete, the lower the CO_2_ penetration [13,25,26]. In cement-based materials, CO_2_ curing makes the microstructure denser, decreases the necessary curing time, decreases permeability and improves mechanical properties [26,27,28].

The use of recycled aggregates from CDW in cement-based materials is a viable alternative to reducing the environmental and economic impact in the construction sector. Although it is difficult to measure the current world production of CDW, it is estimated that it exceeds 3.1 × 10^9^ tonnes per year [29]. Therefore, the use of these recycled aggregates in new constructions would contribute to reducing the impact of CDW in landfills and close the life cycle of these products, which is a clear example of promoting the new circular economy paradigm. There are two major types of recycled aggregates from CDW: recycled concrete aggregates (RCA) composed mainly of concrete particles and unbound aggregates (≥90%) and mixed recycled aggregates (MRA) that can contain up to a maximum of 30% ceramic particles [30]. Recycled aggregates from CDW (RA) are rougher and have higher porosity than natural aggregates (NA), so the particle density would be lower and water absorption higher [20]. The presence of micro-cracks and lower resistance to fragmentation is also one of the properties of recycled aggregates; these differences between RA and NA are mainly due to the attached cement paste. The replacement of NA by RA in cement-based materials generally reduces their mechanical and durability properties [31,32] and the rheological properties are also affected by the substitution of NA with RCA [33]. However, in many cases this behaviour may be due to the method of substitution used. The authors have successfully used different recycled aggregates in previous studies [20,34,35,36].

There are two possibilities for using CO_2_ treatment to improve the properties of cement-based materials made with RA: (i) pre-treatment of recycled aggregates with CO_2_ and (ii) accelerated carbonation of fresh cement-based mixtures. Although the carbonation mechanisms in both situations are similar, some difficulties arise in the latter. In the case of RA, pre-treatment with CO_2_ increases the density and decreases the crushing value, water absorption and porosity. It also improves the interfacial transition zone between the carbonated RA and the cement paste, improving the mechanical properties of concrete made with pre-treated RA [13,17,24,37,38,39,40,41,42,43].

For concrete or mortar made with RA, accelerated carbonation reduces the necessary curing times to reach a certain mechanical strength, water absorption and shrinkage, as well as increasing density and improving mechanical properties. The main problem with accelerated carbonation is that it depends on CO_2_ penetrating through the pores of the concrete matrix, which, as it becomes denser, hinders the entry of CO_2_ and the carbonation of the entire mass. Hence, the porous mixtures, where the entire mass is accessible to CO_2_, are more suitable for applying new accelerated carbonation technologies [13,17,24,38,39,40,41,42,43].

Moreover, the use of porous concrete in pavements allows the infiltration of rainwater, reduces the runoff flow and the damage caused by floods, as well as favours the recharge of aquifers and the growth of trees in cities. Because of this, porous pavements are a very good alternative to mitigate the effects of climate change.

This article studies the effect of accelerated carbonation on the physical–mechanical and chemical properties of vibro-compacted porous concrete for the manufacture of eco-friendly precast elements made with the main types of recycled aggregates from CDW (RCA and MRA). It can be divided into three sections: characterisation of the starting materials, the study of the dry bulk density, water absorption, accessible porosity for water and compressive strength of hardened concrete and the carbon capture of the different mixtures and curing methods.

Due to the great porosity of this vibro-compacted porous concrete, this study has an added advantage, which is that of a draining property of the runoff from rainfall. In recent years, we have seen more torrential rains due to the effects of climate change, so it would help the evacuation of water more quickly and in an environmentally friendly manner, as we could contribute to the recharge of underground aquifers under our roads made with this porous material.

There are currently no CO_2_ capture studies with non-structural porous vibro-compacted concrete made with recycled aggregates from construction and demolition at 28 days of curing, measuring the amount of CO_2_ sequestered by the concrete using the DTA/TGA technique.

## 2. Materials and Methods

### 2.1. Materials

Three types of natural aggregates were used: (i) natural aggregate 0/3 (NA-0/3), (ii) natural gravel 5/7 mm (GN-5/7) and (iii) natural gravel 4/12.5 (GN-4/12.5). Two types of recycled aggregates from CDW obtained from a recycling plant located in Cordoba (Spain) were used: (i) recycled concrete aggregate 2/12.5 (R1) and (ii) mixed recycled aggregate 2/12.5 (R2). The cement used was CEM II/A-L 42.5 R (UNE-EN 197-1: 2011 [44]). A superplasticiser called BASF GLENIUM 3030 NSS with a density of 1210 kg/m^3^ was also used.

Table 1 shows the dry particle density and water absorption of the aggregates calculated in accordance with the standard UNE-EN 1097-6:2013 [45], The cement had a dry bulk density of 2.89 g/cm^3^ according to the data provided by the manufacturer.

### 2.2. Mix Design

Three mixtures of porous concrete (PC) were tested: (i) reference, where only natural aggregates were used (PC-REF); (ii) total replacement of GN-5/7 and GN-4/12.5 for recycled aggregate R1 (PC-R1), and (iii) total replacement of GN-5/7 and GN-4/12.5 for recycled aggregate R2 (PC-R2). A volumetric substitution was made and the replacement was carried out according to Equations (6) and (7). Table 2 shows the three types of porous concrete mixtures tested.

(6) $$W_{R1}=\frac{\rho_{R1}\cdot W_{GN-4/12.5}}{\rho_{GN-4/12.5}}+\frac{\rho_{R1}\cdot W_{GN-5/7}}{\rho_{GN-5/7}}$$

(7) $$W_{R2}=\frac{\rho_{R2}\cdot W_{GN-4/12.5}}{\rho_{GN-4/12.5}}+\frac{\rho_{R2}\cdot W_{GN-5/7}}{\rho_{GN-5/7}}$$

where $W_{R1}$ and $W_{R2}$ are the weights of the recycled aggregates R1 and R2, respectively, in kg/m^3^, used in each of the mixtures, $\rho_{R1}$ and $\rho_{R1}$ are the dried particle densities in g/cm^3^ of the aforementioned aggregates and $W_{GN-5/7}$ and $W_{GN-4/12.5}$ are the weights of the natural aggregates to replace.

The mixtures were made by pouring dry materials from largest to smallest particle size. Later, the saturation water of the aggregates was added and was left for 10 min so that the greatest amount of water possible was absorbed, then the cement and the effective water with the additive were poured. Moulds of 10 cm × 10 cm × 10 cm were used and the concrete mixture was placed in two layers, which were vibro-compacted with a duration of 5 s each layer. The time was adjusted experimentally in the laboratory so that the reference mixture (PC-Ref) had the same density and resistance as the mixture on an industrial scale in the manufacture of draining blocks. This allowed the samples to be demoulded immediately after compaction, just as it is done on an industrial scale.

### 2.3. Hardening Environments

Two types of hardening environments were used:

- Normal climatic chamber (NCC): temperature of 21 ± 2 °C and relative humidity of 65 ± 10%. The concentration of CO_2_ was 0.04% (approximately atmospheric conditions).
- Accelerated carbonation chamber (ACC): same temperature and relative humidity as that for NCC. Only the concentration of CO_2_ was different: 5% CO_2_.

### 2.4. Test Methods

#### 2.4.1. X-ray Diffraction (XRD) Pattern

Raw materials (aggregates and cement) and hardened concrete samples were characterised by X-ray diffraction (XRD). Bruker D8 Discover A25 equipment with CuKα (λ = 1.54050 Ȧ; 40 Kv; 30 mA) was used. Diffraction patterns were measured between 10° and 70° (2θ) at a rate of 0.006 θ min^−1^. In hardened concrete samples, the XRD pattern was analysed at 28 days. The hardened samples were ground to a powder to obtain a representative sample. The samples were immersed in ethanol for the desired curing age for 48 h, in order to stop the hydration reactions of the cement at that age of study [20,46,47].

#### 2.4.2. X-ray Fluorescence Spectrometry (XRF) Analysis

To determine the elemental chemical composition of the raw materials, a wavelength dispersive X-ray fluorescence spectrometry (XRF) analysis was carried out with a power of 4 kW and a ZSX PRIMUS IV (Rigaku).

#### 2.4.3. Thermogravimetric Analysis and Differential Thermal Analysis (TGA-DTA)

Thermogravimetric analysis (TGA) and differential thermal analysis (DTA) were performed for raw materials and hardened concrete samples at 28 days of curing. TGA was performed on a Setaram Setsys Evolution 16/18 apparatus at a heating rate of 5° min^−1^. The test temperature ranged from room temperature to approximately 1000 °C. The TGA indicates the mass at each temperature and the DTA indicates the differential thermal analysis. Differential thermal analysis (DTA) measures endothermic and exothermic transitions as a function of temperature. This is useful for understanding the temperature range in which significant phase changes occur for inorganic compounds. With TGA, we can observe the variability of mass as a function of temperature.

#### 2.4.4. Carbonation Depth

The carbonation depth was determined by spraying a freshly divided surface with phenolphthalein (1 g phenolphthalein, 70 mL ethanol and 100 mL distilled water) according to the standard UNE-EN 14630:2007 [48]. The carbonation depth was measured at 28 days for all hardened samples. Two depths were measured for each sample (one for each half prism).

#### 2.4.5. Compressive Strength of Hardened Specimens

The compressive strength (CS) was measured in accordance with the European standard UNE-EN 12390-3:2020 [49] at the curing ages of 1, 3, 7, 14 and 28 d. A servo-controlled universal testing machine (Ibertest, Mod: MEH-2000/LCW, Madrid, Spain) was used to apply a load at constant speed (300 N/s for CS). Three samples per mixture were tested.

#### 2.4.6. Physical Characterisation of Hardened Samples

The determination of the water absorption, dry bulk density and water-accessible porosity of the concrete was carried out in accordance with the European standard UNE 83980:2014 [50].

## 3. Results and Discussion

### 3.1. Characterisation of Raw Materials

**Figure 1:**
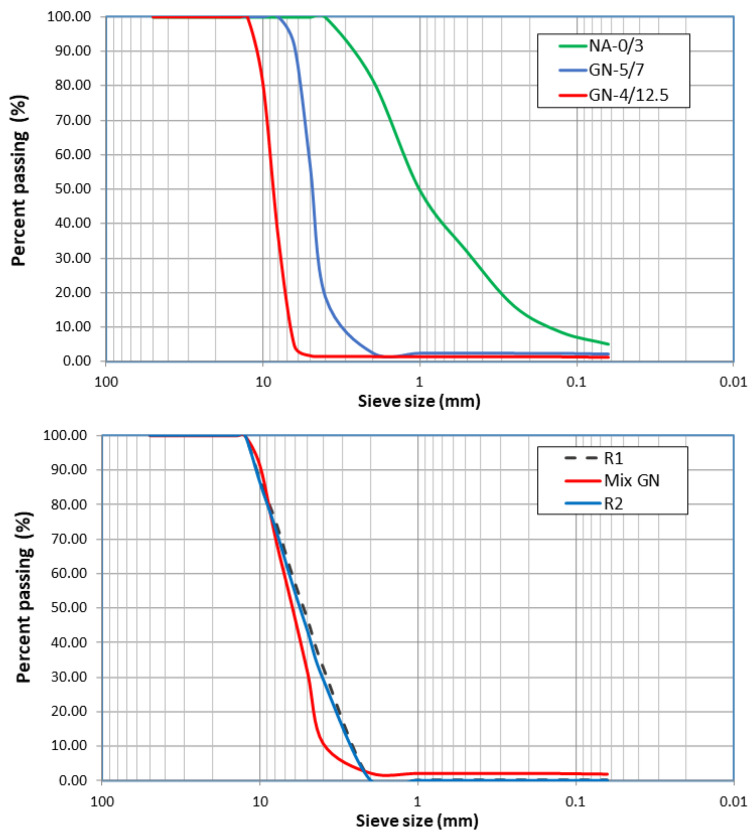
Particle size of natural aggregates, recycled aggregates and the reconstruction of the particle size used in the different mixtures.

A particle size distribution analysis was carried out according to the European standard UNE-EN 933-1:2012 [51]. The first graph of Figure 1 shows the particle size distribution of the three natural aggregates used (NA-0/3, GN-5/7 and GN-4/12.5). To replace the natural aggregates with recycled aggregates, the particle size distribution of the proportions indicated in Table 2 of natural coarse aggregates (GN-5/7 and GN-4/12.5) is represented by Mix GN in the second graph of Figure 1. Both recycled aggregates (R1 and R2) were sieved by 2/12.5 mm fractions. The particle size distributions of R1 and R2 sieved by 2/12.5 mm and Mix GN are practically the same.

Table 3 shows the results of XRF for the raw materials. For cement, CaO was the highest oxide. This result is in accordance with several authors [36,52,53]. The majority composition for the natural aggregates (GN-4/12.5, GN-5/7 and NA-0/3) was CaO (calcium oxide), which is consistent with the calcareous nature of these aggregates (gravel and sand). Unlike native aggregates, in the recycled aggregates from CDW (R1 and R2), the majority composition is SiO_2_ and CaO, although it should be noted that the amount of SiO_2_ is significantly higher than that of CaO, which may be due to the nature of the sands or coarse sand used for the manufacture of mortar or concrete from which this waste comes. These values are consistent with those obtained by Suescum et al. [20]. It can be seen that for R2, there is an increase in the amount of SiO_2_ and Al_2_O_3_ with respect to R1. This is due to the incorporation of the ceramic particles that make up this aggregate, given that the manufacture of the ceramic material contains a large amount of silica (SiO_2_) and alumina (Al_2_O_3_) [54].

**Figure 2:**
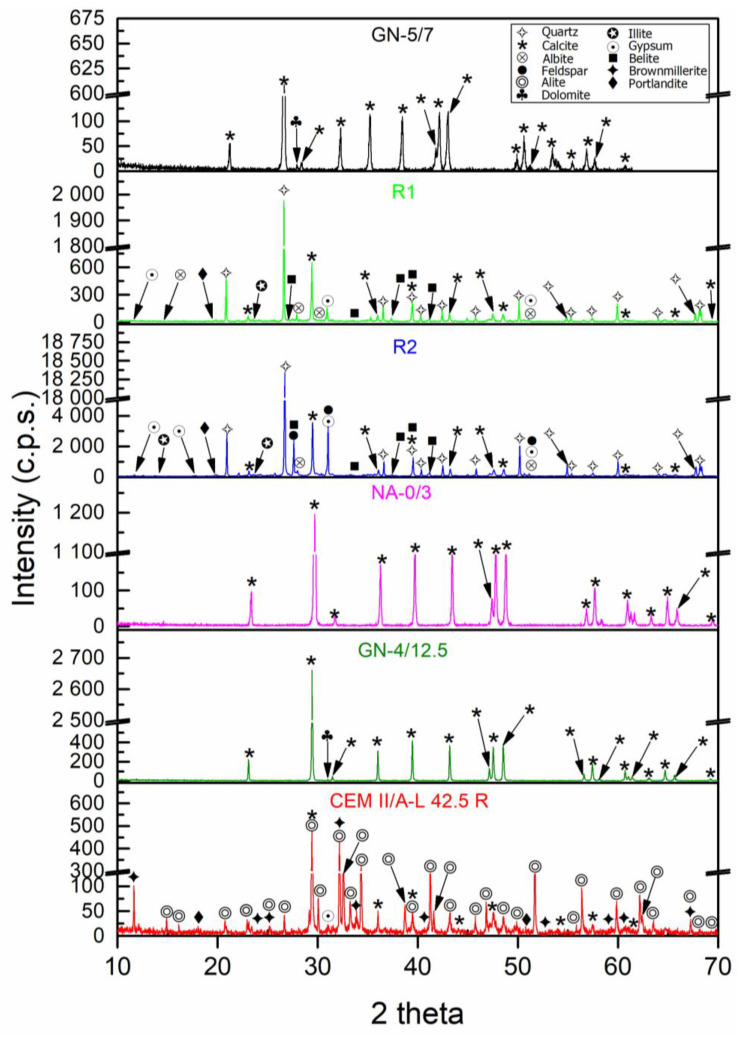
X-ray diffraction patterns of raw materials used for the different mixtures.

The X-ray diffraction patterns of the raw materials used for this study are shown in Figure 2. Due to the limestone nature of GN-5/7, GN-5/12.5 and NA-0/3, the phase found in these materials is calcite (CaCO_3_) (05-0603) [55]. It was also observed that for the larger aggregates (GN-5/7 and GN-5/12.5), there was a secondary phase identified, which was dolomite (36-0426) [55]. For aggregates from CDW (R1 and R2), the main phase found was quartz (SiO_2_) (05-0490) [55], while other phases found in this material with high intensities, although not as high as quartz, included calcite (CaCO_3_) (05-0586) [55], and other minority phases shown were of albite (Na(Si_3_Al)O_8_) (10-0393) [55], illite K(AlFe)_2_AlSi_3_O_10_(OH)_2_·H_2_O (15-0603) [55], belite(Ca_2_SiO_4_) (09-0351), portlandite (44-1481) and gypsum (21-0816) [55]. In addition to these compounds, feldspar (AlSi_3_O_8_) (89-8574) [55] is also present, although in a very minor form. The presence of belite may be due to the remains of cement found in R1 that have not hydrated [20]. The phases detected for the cement were alite (C_3_S)(86-0402), brownmillerite (11-0124) [55], calcite (05-0586) [55], gypsum (21-0816) [55] and portlandite (44-1481) [55], which is an expected result for a cement [44]. These results are in accordance with other authors [20,56,57,58] and correlate with the chemical composition in Table 3.

**Figure 3:**
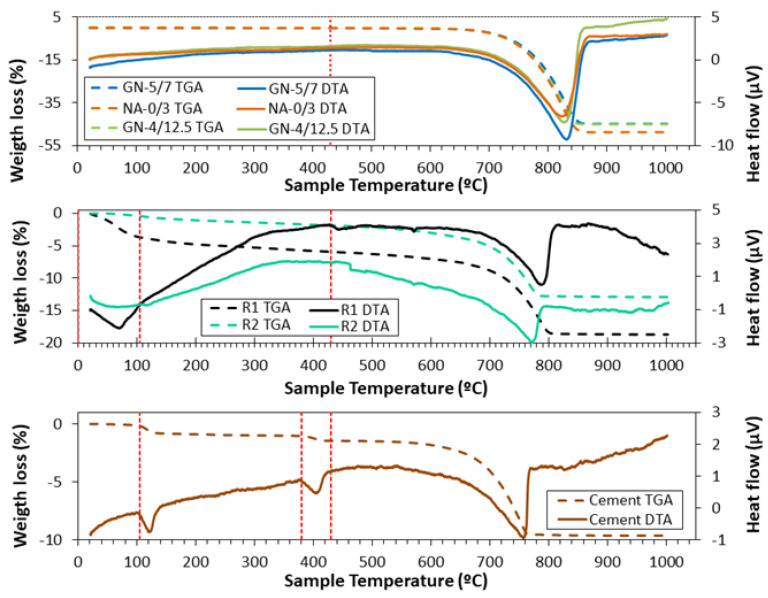
Thermogravimetric analysis (TGA) and differential thermal analysis (DTA) for starting materials. TGA (solid line) and DTA (dotted line).

Figure 3 shows the thermogravimetric analysis (TGA) and differential thermal analysis (DTA) for the raw materials used in this study. Different stages can be observed depending on the material analysed. For the natural aggregates (NA-0/3, GN-4/12.5 and GN-5/7), it was observed that the results were similar, since according to XRD and XRF, their main composition was the same (calcite), and it must be taken into account that in these materials we would only have one phase, which is the decomposition of calcite (from 430° to 1000°), according to Equation (8):

(8) $${CaCO}_{3}\to CO_{2}+CaO$$

For aggregates from CDW (R1 and R2), three sections were identified: (i) the loss of moisture that can be seen from room temperature to 105 °C; (ii) the loss of aluminates and calcium silicates from 105 °C to 430 °C; (iii) and the last section, which is the decomposition of calcium carbonate (according to Equation (8)) that ranges from 430 °C to 1000 °C. For R2, a peak at 120 °C (between 105 °C and 130 °C) was observed, which is due to the decomposition of gypsum, identified by XRD [59]. In addition, a delay in weight loss was observed in R2 compared to R1, with the endothermic peak showing a more pronounced delay. This is due to the lower amount of calcite R1 and the clear presence of illite and albite, which decomposes at a lower temperature (about 660 °C) than calcite.

In TGA/DTA of cement, four stages can be observed: (i) from room temperature to 105 °C, which corresponds to the elimination of moisture; (ii) from 105 °C to 380 °C, dehydration of aluminates and calcium silicates takes place [36,53]; (iii) decomposition of portlandite from 380 °C to 430 °C (identified by XRD) [36,53] due to this decomposition, an endothermic peak appears at 410 °C; (iv) calcite decomposition [58,60] occurs between 430 °C and 1000 °C according to Equation (8).

### 3.2. Dry Bulk Density, Water Absorption and Accessible Porosity for Water

**Figure 4:**
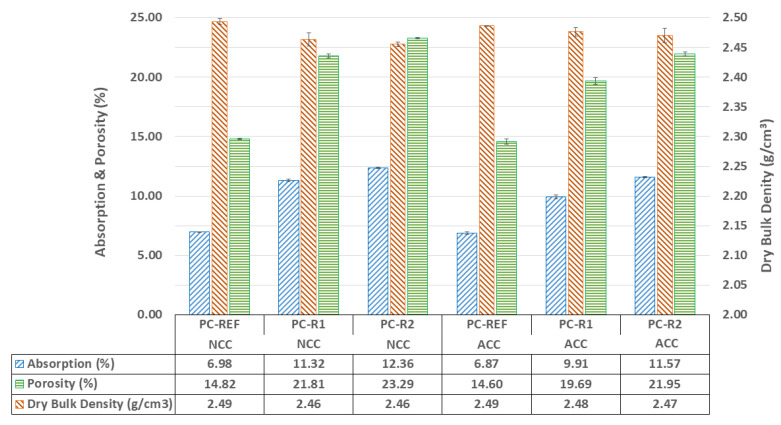
Dry bulk density, water-accessible porosity and absorption for the different mixtures at a curing age of 28 d for the different curing environment regimes.

Figure 4 shows the results of water absorption, accessible porosity for water and dry bulk density at the curing age of 28 d.

As shown in Figure 4, the mixes made with RA (PC-R1 and PC-R2) obtained higher water absorption and lower dry bulk density than reference concrete made with NA (PC-RER), which may be due to the higher porosity of the RA [61,62]. Recycled aggregates (R1 and R2) showed a lower particle dry bulk density and higher water absorption than NA (Table 1), which is an intrinsic property of recycled aggregates from CDW [20,63,64,65].

Accelerated carbonation in the ACC environment on the PC-REF samples slightly decreases the accessible porosity for water and the percentage of water absorption, despite maintaining the dry bulk density, which may be because the penetration of CO_2_ in this mixture is more complicated due to the lower porosity of the aggregates that compose its mixture [20,38,40].

Regarding the effect of curing in a CO_2_ environment on the specimens made with RA (PC-R1 and PC-R2), it was observed that the dry bulk density values increased and that the water absorption and accessible porosity for water increased. The lowest dry bulk density obtained is for the PC-R2 mix, which also leads to the highest porosity. These results are in accordance with previous studies on the use of recycled masonry aggregates for the manufacture of concrete blocks for masonry mortars [66]. This is due to the densification that occurs in the samples cured with CO_2_ (see Figure 4), caused by the appearance of calcium carbonate (CaCO_3_) during the carbonation process, shown in Equations (1)–(5). The appearance of this compound fills the pores of the hardened sample, increasing the dry density and decreasing the porosity [20,38,40].

### 3.3. Compressive Strength

**Figure 5:**
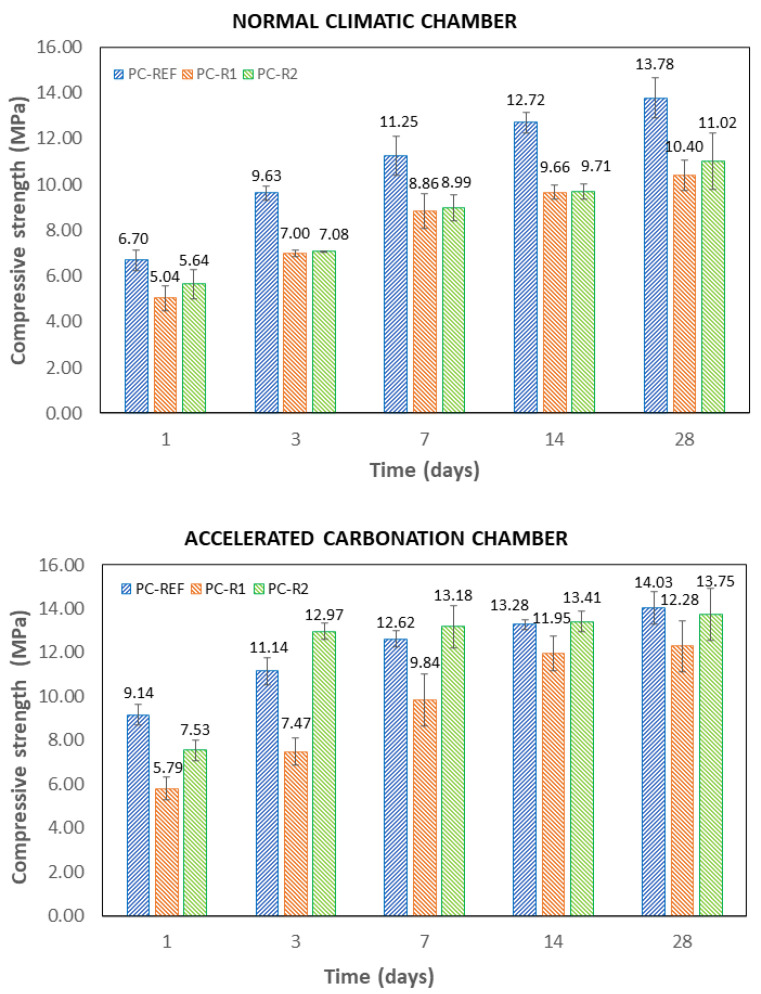
Compressive strength at 1, 3, 7, 7, 14 and 28 days for the different curing environments (ACC and NCC).

Figure 5 shows the mean and dispersion of the compressive strength values of the different mixtures studied in both curing environments. The mean compressive strength values for all samples and curing environment increased over time, which is a typical response of Portland cement-based materials [67,68,69,70,71].

In the NCC curing environment, mixes made with RA (PC-R1 and PC-R2) at all curing ages showed lower compressive strength mean values than the reference mixture (PC-REF), even though the substitution was made using the volumetric method that usually gives better results than substitution by weight [72,73,74,75,76,77,78]. This is in accordance with other studies [20,36,79] due to RA from CDW having poorer mechanical properties compared to natural aggregates. The compressive strength mean values of PC-R1 were reduced by an average of 24.4% (range 21–27%) with respect to PC-REF and in the case of PC-R2 the average reduction was 21.2% (range 16–26%). In both cases, the mechanical strength drop percentages were not affected by the curing time.

In the case of the ACC environment, the average value of the compressive strength of PC-R1 was reduced by an average of 22.6% (range 10–36%), although in this case, the curing time significantly affected this percentage of reduction. The difference was higher at early stages and lower after 14 days of curing. On the contrary, the PC-R2 mixture showed a reduction in compressive strength of 18% compared to PC-REF with one day of curing, but its mechanical strength evolved very favourably over time, with PC-R2 showing compressive strength mean values slightly higher than PC-REF at 3, 7 and 14 days of curing and similar values at 28 days.

The mean compressive strength values of the PC-REF mixtures significantly increased under accelerated carbonation conditions. The mechanical strength increased by more than 36% at one day of curing, which can be beneficial for the precast industry since they can reduce the curing time products and productivity of the factory, lower energy consumption, or even reduce the amount of cement and carbon footprint of the precast element [67]. In the mixture made with aggregate R1 (PC-R1), the mechanical strength also increased with CO_2_ curing.

For the mixture made with R2 aggregate (PC-R2), the effect of carbonation appeared more prominent at 3, 7 and 14 days, even exceeding the compressive strength mean values of the mixtures made with NA (PC-REF). Similar conclusions were found by Suescum et al. in other studies carried out using mortars made with mixed recycled aggregates [20,22,26]. This increase in compressive strength in CO_2_ curing environment could be due to the carbonation of the mixture and thus to the slight densification of the mixture, as can be seen in Figure 4.

Although the mean compressive strength values were generally reduced with the use of recycled aggregates from CDW, it was observed that curing in a CO_2_ environment was more effective when using this type of aggregate, which can be explained by the fact that the particles of aggregates R1 and R2 are more porous than NA (Table 1) and have a higher specific surface area in contact with the cement and gases [71].

Finally, the highest increases in the CO_2_ environment occurred early—1, 3, and 7 days. This growth in mechanical strength properties at these early stages can be observed, especially for PC-R2/ACC, where it reaches an increase of more than 72%. This is due to the fact that at shorter curing times, the portlandite from the cement reacts with CO_2_, forming calcium carbonate (Equations (1)–(5)). However, the percentage of growth in mechanical strength decreases at later stages, as can be observed for PC-R2/ACC, where the increase between day 14 and day 28 was less than 3%. This can be attributed to the calcium carbonate formed during the first days of curing with high concentrations of CO_2_ densifying the pores and preventing the entry of CO_2_ and the consequent carbonation reactions in the internal concrete matrix. Similar conclusions were drawn by Pingping et al. [78].

### 3.4. XRD of Hardened Mixtures

**Figure 6:**
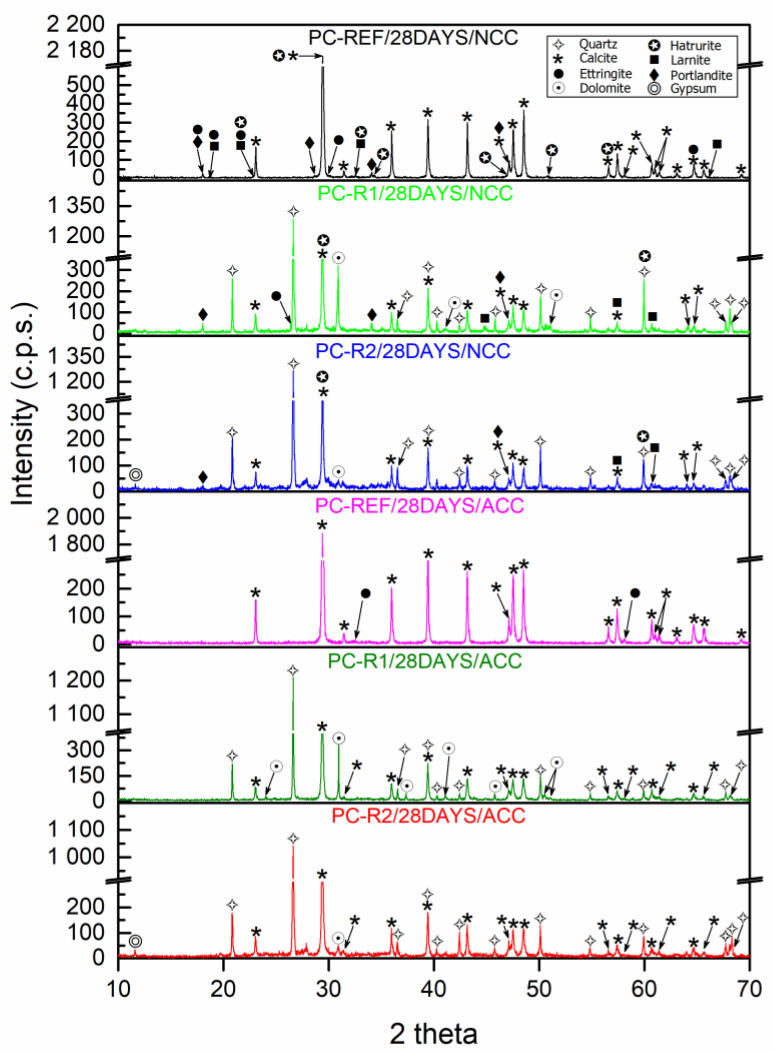
X-ray diffraction patterns of hardened concrete at 28 days of curing in NCC and ACC environment.

Figure 6 shows the XRD patterns obtained for the PC samples cured in ACC and NCC at 28 days.

For the PC-REF/28DAYS/NCC mix, one main phase was identified as calcite (05-0586) [55], which is in accordance with the nature of the aggregates used in this mix. As other secondary phases, ettringite (02-0059) [55], portlandite (441881) [55], belite (09-0351) [55] and alite (86-0402) [55] were identified.

However, for the PC-REF/28DAYS/ACC mixture, a majority phase was identified as calcite (05-0586) [55] and a very minor phase was identified as ettringite (02-0059) [55], which shows the complete carbonation of the mixture, due to the densification of the internal structure. These results were similar to those found by Suescum et al. in their study of mortars made with MRA [20].

For the samples made with R1 (PC-R1), the same main phases were identified in both ACC and NCC, quartz (05-0490) [55], calcite (05-0586) [55] and dolomite (36-0426) [55]. However, other secondary phases were identified in the mixture cured in the normal climatic chamber (NCC), which were portlandite (44-1881) [55], alite (86-0402) [55], belite (09-0351) [55] and ettringite (02-0059) [55], due to the densification of the sample during curing in the NCC environment as a result of the carbonation of the mixture.

For the samples with R2 aggregate (PC-R2), the same main phases were identified in both ACC and NCC as occurred with the mixture made with R1 aggregate: quartz (05-0490) [55], calcite (05-0586) [55] and dolomite (36-0426) [55]. Other secondary phases were also identified in the mixture cured in the normal climatic chamber (NCC), which were portlandite (44-1881) [55], alite (86-0402) [55] and belite (09-0351) [55]. In both curing environments, the presence of the gypsum phase (21-0816) [55] was detected, which is in accordance with the XRD carried out on the raw material, where this same phase was observed in the R2 aggregate.

### 3.5. Depth of Carbonation

**Figure 7:**
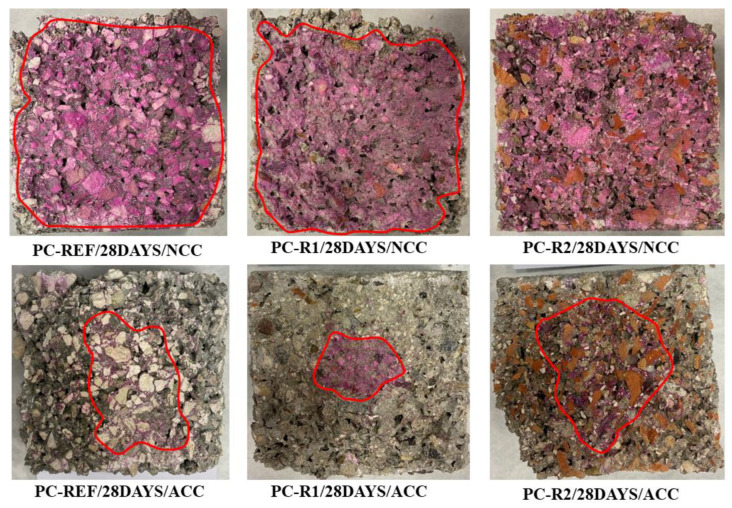
Carbonation depth of the different mixtures at 28 days of curing in NCC and ACC environment.

Figure 7 shows the carbonation depth of the different mixtures at 28 days of curing in the NCC and ACC environments. The purple indicates the part of the mixture that has not carbonated, and the whitish area the part that has.

It is observed that for samples PC-REF/28DAYS/NCC and PC-R1/28DAYS/NCC, there was an average carbonation depth of 3 and 4 mm, respectively, while for sample PC-R2/28DAYS/NCC, no carbonated part was observed.

Samples PC-REF/28DAYS/ACC, PC-R1/28DAYS/ACC and PC-R2/28DAYS/ACC had an average carbonation depth of 23.5, 34.6 and 22.5 mm respectively.

Therefore, it can be said that the substitution of NA for RCA favours carbonation, due to the increased porosity of the RCA aggregate as opposed to the lower porosity of NA, which contributes to greater CO_2_ sequestration and therefore an increase in mass from the densification of the aggregate and therefore of the mixture [13,61,80].

It can also be observed that in the mixtures PC-REF/28DAYS/ACC and PC-R2/28DAYS/ACC, the carbonation fronts are irregular due to the irregularity of the interconnected pores, so that in addition to this qualitative test, it is convenient to carry out TGA/DTA analyses and to account for the carbonated part in a more exhaustive way.

### 3.6. TGA/DTA of Hardened Mixtures

**Figure 8:**
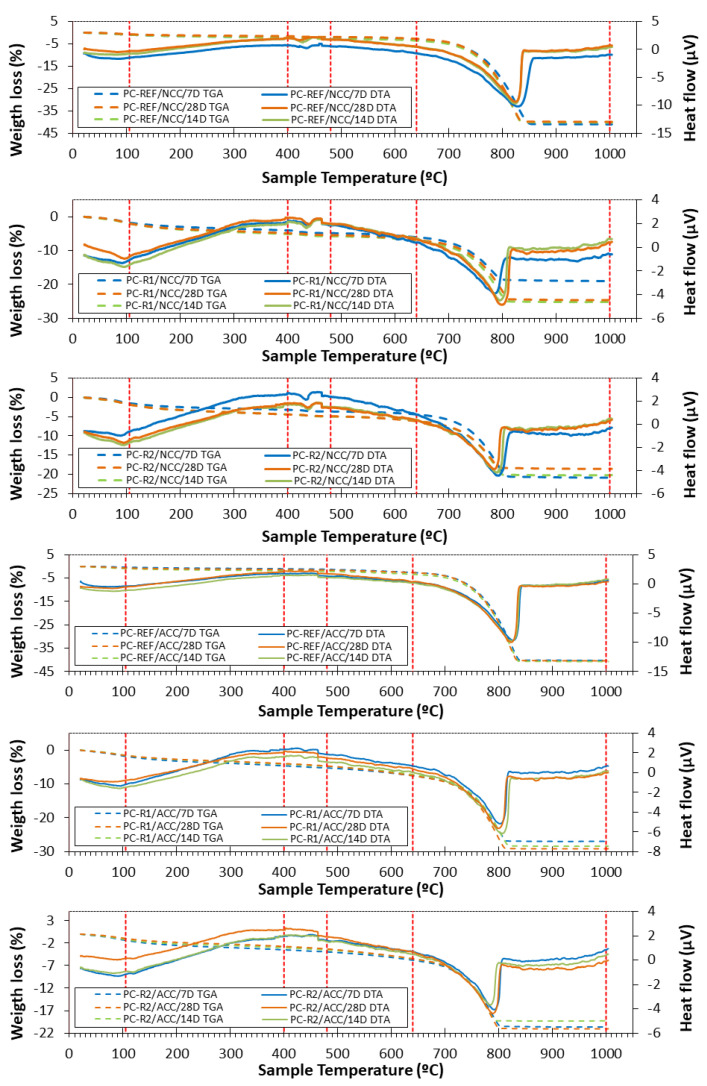
TGA and DTA of the different mixtures at 7, 14 and 28 days of curing in NCC and ACC environment.

Figure 8 shows the different graphs of the TGA (solid lines) and DTA (dashed lines) of the different mixtures at 7, 14 and 28 days of curing in the NCC and ACC environments.

A five-stage division was observed:

(i) **From room temperature to 105 °C**. In this phase, the loss of physically absorbed water was observed, i.e., moisture was lost. The mixtures PC-R1 and PC-R2 showed a higher moisture content related to the higher water absorption presented by R1 and R2, observable on practically all the curing days, since the DTA peak is higher than for the reference simples [20,47].
(ii) **In the second stage, from 105 °C to 400 °C**, dehydration of the calcium silicates and aluminates took place, also according to the analyses carried out on the raw materials previously used [53].
(iii) **In the third stage, from about 400 to 480 °C,** the decomposition of portlandite occurred, which was identified by an endothermic peak at around 410–420 °C. For the case of the reference mixture, a peak was found for all curing times in the normal climatic environment. However, for this same mixture in the CO_2_ curing environment the portlandite disappeared for all the curing times studied. This indicates that the carbonation is complete as indicated by XRD. This is also in accordance with the observed improvements in compressive strength (see Figure 5). For the mixtures PC-R1 and PC-R2, cured in the NCC environment, portlandite was also observed for all curing times studied. However, for these same mixtures cured in the ACC, the portlandite practically disappears for the curing times analysed. The difference that exists to be able to observe the peak of the portlandite at the minimum curing age studied (7 days) is due to the amount of this compound in the mixture [81].
(iv) **The fourth stage, ranging from 480 to 640 °C,** comprised the section where weight loss occurred due to the initial decomposition of carbonates formed during the hardening process [82].
(v) Finally, **in the fifth and last stage from 640 to 1000 °C**, decomposition of calcium carbonate occurred [83].

Once all the TGA/DTA analyses of the hardened samples for both curing environments were shown, the amount of CO_2_ sequestered (as a percentage) by the different samples, curing environments and days of curing were calculated from the TGA/DTA analysis. A summary of the results can be seen in Table 4. These results determine the percentage mass loss variation corresponding to each phase previously found in the TGA, thus attributing each percentage mass loss to each compound that formed the mixture. Once these mass losses are attributed to the compounds, the difference between the mass loss associated with calcium carbonate between the carbonation chamber and the normal climatic chamber corresponds to the CO_2_ absorption according to Equation (9) This is because when comparing two equal samples, in which the only variant from one chamber to the other is the CO_2_ concentration, it is obvious that the difference corresponds to the sequestered CO_2_, shown as a product of the carbonation reaction.

(9) $$Sequestration\;{CO}_{2}=\%\;CaCO_{3}\;(ACC)-\%\;CaCO_{3}\;(NCC)$$

Mass losses between 105 °C and 400 °C were assigned to the setting water of the cement, which is the water of the hydrated calcium silicates and aluminates. Losses between 400 °C and 480 °C were attributed to portlandite. Finally, the fourth and fifth TGA/DTA stages were unified to quantify the decomposition of CaCO_3_, which is shown in Table 4 in the column corresponding to calcium carbonate.

The amount absorbed by the samples cured in the NCC environment has been considered a reference, although in reality a small sequestration would occur, which for practical purposes we consider negligible because the amount of CO_2_ in the environment can be considered almost zero. For the PC-REF sample, the amount absorbed was 1.6 kg CO_2_/t (0.1592%) at curing of 7 days. The maximum capture capacity of the PC-REF sample was at 28 days, with a value of 12.37 kg/t CO_2_. The large difference in this sample between the curing for 7 and 28 days is noteworthy, with an increase of 700%, with this material serving as a CO_2_ sink.

For all curing ages, the PC-R1 mixture had the highest absorption. This absorption increased in turn with the age of curing, obtaining at 28 days an absorption of 52.52 kg/t, the maximum value recorded among all the mixtures and duration of curing. For this mixture, there is an increase of 30% when compared to the 7-day stage, where absorption value was 40.48 kg/t. Compared to the mixture PC-REF, it obtained between 240% and 333% more absorption, depending on the curing stage.

The PC-R2 sample is the one that obtained the worst data, reaching 5.5 kg/t after 28 days of curing, lower than the value obtained by the other two mixtures, leading to the conclusion that the ceramic material has worse CO_2_ capture properties than the concrete waste due to the cement paste adhered to it.

Accelerated carbonation increased the CO_2_ sequestration capacity compared to the normal chamber by 123%, 525%, and 55% for the PC-REF, PC-R1 and PC-R2 mixtures, respectively.

It is therefore clear that curing in an ACC environment means a great increase in CO_2_ absorption, obtaining values of 52.52 kg/t, which is an increase of 525% with respect to curing in an NCC environment. If we add to this the use of RCA, we also obtain improvements of 333% with respect to the mixture composed of natural aggregates. These results reveal that accelerated carbonation of cement-based products containing recycled aggregates is a promising technology for CO_2_ capture and utilisation, making CO_2_ a potential industrial feedstock.

## 4. Conclusions

This study on the effect of accelerated carbonation on the physical–-mechanical and CO_2_ capture of a porous vibro-compacted concrete for use in precast products that incorporate two types of recycled aggregates from construction and demolition waste, yielded the following conclusions:

- The substitution of recycled concrete aggregate (R1) presented a very significant CO_2_ sequestration advantage.
- Accelerated carbonation reduced the curing time (setting of the mixture in less curing time), reduced water accessible porosity, and increased the dry bulk density compressive strength mean values, especially during early stages of curing (1–14 days).
- In vibro-compacted concrete samples subjected to accelerated carbonation, a decrease or disappearance of the portlandite phase and an increase in calcium carbonate was observed using TGA/DTA and XRD techniques, with the consequent sequestration of CO_2_.
- The amount of CO_2_ sequestration at 28 days for the PC-REF (natural aggregates), PC-R1 (recycled concrete aggregates) and PC-R2 (mixed recycled aggregates) mixtures was approximately 12.36 kg/t, 52.52 kg/t and 5.5 kg/t, respectively, representing an increase of 123%, 525%, and 55% over the curing under an atmospheric environment.

This study indicates that accelerated carbonation of porous vibro-compacted concrete containing recycled concrete aggregates and mixed recycled aggregates is a promising technology for CO_2_ capture and utilisation in precast products. The main advantages for the precast concrete industry would be, among others, the significant improvement in the mechanical performance of the cement-based materials, reduction in curing times, reduction in the amount of cement to be used to achieve similar mechanical strength in CCN curing conditions, and a lower carbon footprint per cubic metre of concrete. In addition to this, the use of recycled aggregates from construction and demolition waste would promote the new circular economy paradigm and the CO_2_ sink effect of precast concrete elements, which could mitigate climate change.

## Figures and Tables

**Table 1:** Dry particle density and water absorption.

Material	Dry Particle Density (g/cm^3^)	Water Absorption (%)
Natural aggregate (NA-0/3)	2.62	1.78
Natural gravel (GN-5/7)	2.43	2.64
Natural gravel (GN-4/12.5)	2.47	3.14
Recycled aggregate (R1)	2.21	7.43
Recycled aggregate (R2)	2.17	9.03

**Table 2:** Dosage of vibro-compacted concrete samples (per cubic metre).

Materials	PC-REF (kg/m^3^)	PC-R1 (kg/m^3^)	PC-R2 (kg/m^3^)
CEM	240.00	240.00	240.00
GN-4/12.5	800.00	-	-
NA-0/3	500.00	500.00	500.00
GN-5/7	900.00	-	-
R1	-	1535.74	-
R2	-	-	1504.69
Absorption water *	57.74	122.96	143.59
Effective water	96.00	96.00	96.00
Total water	153.74	218.96	239.59
w/c **	0.4	0.4	0.4
BASF GLENIUM 3030 NSS	0.60	0.60	0.60

* Aggregate absorption water calculated according to Table 1. ** Effective water/cement ratio.

**Table 3:** X-ray fluorescence (XRF) of the raw materials.

Oxides	Cement	GN-4/12.5	NA-0/3	GN-5/7	R1	R2
Na_2_O	0.24	-	-	-	0.81	0.82
MgO	1.32	0.89	0.64	0.96	2.77	3.15
Al_2_O_3_	3.73	0.20	0.78	0.73	7.77	10.79
SiO_2_	15.58	0.39	1.76	2.13	51.41	52.15
P_2_O_5_	0.10	-	-	-	0.11	0.12
SO_3_	4.80	0.07	0.09	0.11	1.13	1.37
Cl_2_O_3_	0.18	-	-	0.05	0.06	0.12
K_2_O	1.21	0.04	0.15	0.09	1.79	2.43
CaO	70.02	98.32	92.09	95.76	30.62	24.55
TiO_2_	0.22	-	-	-	0.43	0.55
MnO_2_	0.06	-	-	-	0.09	0.09
Fe_2_O_3_	2.45	0.09	4.50	0.18	2.75	3.67
CuO	-	-	-	-	-	-
ZnO	0.03	-	-	-	-	-
SrO	0.08	-	-	-	0.03	0.04
Rb_2_O	-	-	-	-	-	-
Cr_2_O_3_	-	-	-	-	0.21	0.15

**Table 4:** CO_2_ sequestration results for each mixture in the different curing environments for curing of 7, 14 and 28 days.

Mixtures	ΔMass (%)				H_2_O Cement	Portlandite	CaCO_3_	CO_2_ Seq	CO_2_ Seq
	105–400 °C	400–480 °C	480–640 °C	640–1000 °C	(%)	(%)	(%)	(%)	(g/t)
PC-REF/7DAYS/NCC	−1.2752	−1.7077	−2.3130	−40.5267	1.2752	0.4325	38.8190	0.1592	1592.4310
PC-REF/7DAYS/ACC	−0.8015	−1.0649	−2.2948	−40.0432	0.8015	0.2635	38.9782		
PC-R1/7DAYS/NCC	−3.1483	−3.9885	−5.1963	−18.3624	3.1483	0.8402	14.3739	4.0476	40,476.3909
PC-R1/7DAYS/ACC	−2.8449	−3.4805	−5.8313	−21.9021	2.8449	0.6356	18.4216		
PC-R2/7DAYS/NCC	−2.3160	−2.7638	−3.5338	−20.2394	2.3160	0.4478	17.4757	0.2415	2415.0101
PC-R2/7DAYS/ACC	−2.1509	−2.6213	−4.4900	−20.3385	2.1509	0.4704	17.7172		
PC-REF/14DAYS/NCC	−1.3326	−1.8820	−2.6694	−39.6945	1.3326	0.5494	37.8125	1.2012	12,012.1098
PC-REF/14DAYS/ACC	−0.9163	−1.1992	−2.4841	−40.2129	0.9163	0.2829	39.0137		
PC-R1/14DAYS/NCC	−3.1467	−3.8229	−5.2193	−23.6197	3.1467	0.6763	19.7968	4.1610	41,610.4493
PC-R1/14DAYS/ACC	−2.6401	−3.2689	−5.7487	−27.2267	2.6401	0.6288	23.9578		
PC-R2/14DAYS/NCC	−2.6237	−3.1832	−4.3868	−18.7149	2.6237	0.5595	15.5316	0.3791	3791.0311
PC-R2/14DAYS/ACC	−1.8525	−2.3550	−4.3778	−18.2658	1.8525	0.5025	15.9107		
PC-REF/28DAYS/NCC	−0.8775	−1.2202	−2.0380	−39.5306	0.8775	0.3427	38.3103	1.2369	12,368.6202
PC-REF/28DAYS/ACC	−0.6853	−0.9253	−2.0187	−40.4725	0.6853	0.2400	39.5472		
PC-R1/28DAYS/NCC	−2.9087	−3.4849	−4.9369	−23.0493	2.9087	0.5761	19.5644	5.2526	52,526.2249
PC-R1/28DAYS/ACC	−2.4322	−3.1562	−6.0719	−27.9732	2.4322	0.7240	24.8170		
PC-R2/28DAYS/NCC	−2.5870	−3.0668	−4.3516	−17.0063	2.5870	0.4798	13.9395	0.5509	5508.6438
PC-R2/28DAYS/ACC	−1.7986	−2.3600	−4.5540	−16.8504	1.7986	0.5614	14.4904		

## Data Availability

Not applicable.
